# 合成酚类化合物的脂代谢干扰效应与致肥胖作用

**DOI:** 10.3724/SP.J.1123.2023.12018

**Published:** 2024-02-08

**Authors:** Huinan LIU, Zhendong SUN, Qian S. LIU, Qunfang ZHOU, Guibin JIANG

**Affiliations:** 1.中国科学院生态环境研究中心,环境化学与生态毒理学国家重点实验室, 北京 100085; 1. State Key Laboratory of Environmental Chemistry and Ecotoxicology, Research Center for Eco-Environmental Sciences, Chinese Academy of Sciences, Beijing 100085, China; 2.中国科学院大学资源与环境学院, 北京 100049; 2. College of Resources and Environment, University of Chinese Academy of Sciences, Beijing 100049, China; 3.国科大杭州高等研究院环境学院, 浙江 杭州 310024; 3. School of Environment, Hangzhou Institute for Advanced Study, University of Chinese Academy of Sciences, Hangzhou 310024, China

**Keywords:** 合成酚类化合物, 肥胖, 环境致肥胖物质, 脂代谢, 综述, synthetic phenolic compounds (SPCs), obesity, environmental obesogen, lipid metabolism, review

## Abstract

随着社会经济的不断发展,肥胖已经成为一种不可忽视的全球性流行疾病。除了受到遗传和饮食因素的影响外,肥胖的形成与发展也与环境中的化学物质暴露有关。研究发现一些具有内分泌干扰活性的化学物质可以影响机体脂代谢,如促进脂肪细胞形成、引起体内脂质累积增加等,这类物质被称为“环境致肥胖物质”。合成酚类化合物广泛用于工业生产和日常生活用品中,如塑料制品、消毒剂、杀虫剂和食品添加剂等,它们可通过饮食、饮水或皮肤接触等方式进入人体,是影响人体健康的潜在威胁。研究显示,合成酚类化合物具有内分泌干扰效应,可以影响脂肪生成过程、干扰脂代谢,具有致肥胖作用。由于这类物质都具有类似的苯酚结构,因此系统梳理这类化合物脂代谢干扰效应与致肥胖作用,有助于理解其构效关系与潜在健康风险。本文阐述了筛选环境致肥胖物质的3种常用研究模型,即体外分子互作研究模型、细胞成脂分化模型和动物脂代谢实验模型,并比较了各种模型的适用场景和优缺点。在此基础上,针对3种合成酚类化合物,即双酚A及其类似物、烷基酚和酚类抗氧化剂,结合其体外细胞与动物实验数据,对这些化合物的暴露现状和脂代谢干扰效应进行了分类描述,从它们对脂肪生成和代谢、肝组织脂代谢的影响等方面展开讨论。基于近年来脂代谢和致肥胖效应的相关研究,本文归纳总结了环境内分泌干扰物干扰脂代谢的内在毒理机制,主要包括核受体调控作用、炎症和氧化应激、肠道微环境、表观遗传和其他信号通路。最后本文指出了当前针对环境污染物,特别是新污染物,影响机体脂代谢引起肥胖效应研究中的不足,并对该领域研究进行了展望。

## Abstract (en)

Given continuous development in society and the economy, obesity has become a global epidemic, arousing great concern. In addition to genetic and dietary factors, exposure to environmental chemicals is associated with the occurrence and development of obesity. Current research has indicated that some chemicals with endocrine-disrupting effects can affect lipid metabolism *in vivo*, causing elevated lipid storage. These chemicals are called “environmental obesogens”. Synthetic phenolic compounds (SPCs) are widely used in industrial and daily products, such as plastic products, disinfectants, pesticides, food additives, and so on. The exposure routes of SPCs to the human body may include food and water consumption, direct skin contact, *etc*. Their unintended exposure could cause harmful effects on human health. As a type of endocrine disruptor, SPCs interfere with adipogenesis and lipid metabolism, exhibiting the characteristics of environmental obesogens. Because SPCs have similar phenolic structures, gathering information on their influences on lipid metabolism would be helpful to understand their structure-related effects. In this review, three commonly used research methods for screening environmental obesogens, including *in vitro* testing for molecular interactions, cell adipogenic differentiation models, and *in vivo* studies on lipid metabolism, are summarized, and the advantages and disadvantages of these methods are compared and discussed. Based on both *in vitro* and *in vivo* data, three types of SPCs, including bisphenol A (BPA) and its analogues, alkylphenols (APs), and synthetic phenolic antioxidants (SPAs), are systematically discussed in terms of their ability to disrupt adipogenesis and lipid metabolism by focusing on adipose and hepatic tissues, among others. Common findings on the effects of these SPCs on adipocyte differentiation, lipid storage, hepatic lipid accumulation, and liver steatosis are described. The underlying toxicological mechanisms are also discussed from the aspects of nuclear receptor transactivation, inflammation and oxidative stress regulation, intestinal microenvironment alteration, epigenetic modification, and some other signaling pathways. Future research to increase public knowledge on the obesogenic effects of emerging chemicals of concern is encouraged.

人体肥胖问题日益凸显,由此给社会公共卫生造成了沉重的负担。根据世界卫生组织统计,1975年以来世界肥胖人数增长近3倍,2016年有39%的成年人被诊断为超重,其中肥胖人数占比13%^[1]^。肥胖作为糖尿病、心血管疾病、癌症等疾病的关键风险因素,严重危害人类健康^[2]^。肥胖是一种多因素疾病,除了遗传因素外,高热量饮食摄入、久坐不动等生活方式被认为是造成肥胖的主要原因^[3]^。然而,越来越多的研究表明,一些环境内分泌干扰物(endocrine disrupting chemicals, EDCs)暴露也可以刺激脂肪细胞分化,引起脂肪组织累积,从而造成机体肥胖^[2,4]^。2006年,Grun和Blumberg将这类EDCs命名为“致肥胖物质(obesogen)”^[5]^。这类化合物大多具有亲脂性,可在脂肪组织中沉积^[6]^,能够增加脂肪细胞的数量和/或大小,干扰脂肪组织代谢,改变食欲、饱腹感和食物偏好等,从而导致肥胖^[7]^。

目前已经发现的环境致肥胖物质有50多种,包括有机锡、邻苯二甲酸酯、双酚A(bisphenol A, BPA)及其类似物、全氟化合物等^[8]^,这些化学品应用广泛,可存在于食品加工、食品包装、化妆品和个人护理产品、家具、电子产品、消毒剂、洗涤剂、杀虫剂/除草剂、塑料制品等一系列生产过程与相关产品中^[8]^。其中,人工合成的一些带有苯酚结构的化合物,如BPA、烷基酚等合成酚类化合物(synthetic phenolic compounds, SPCs),经鉴定具有内分泌干扰活性,可以影响脂代谢,是环境致肥胖物质^[9]^。这类化合物具有类似的化学结构,在脂代谢干扰活性上可能会具有一定的相似性。因此系统梳理这类化合物的脂代谢干扰效应与致肥胖作用,有助于理解外源化合物调控脂代谢影响机体健康的机制。本文围绕包括BPA、烷基酚、酚类抗氧化剂等SPCs,在脂代谢研究模型介绍的基础上,系统综述了它们的环境暴露水平、脂代谢干扰效应与毒理学作用机制。

## 1 脂代谢干扰效应研究模型

目前评价环境污染物的脂代谢干扰效应,主要采用体外分子互作研究模型、细胞成脂分化模型与动物脂代谢实验模型。

### 1.1 体外分子互作研究模型

内分泌核受体如过氧化物酶体增殖物激活受体(PPARα、PPARγ)、维甲酸X受体(RXR)、雌激素受体(ER)、雄激素受体(AR)、甲状腺激素受体(TR)、糖皮质激素受体(GR)等作为转录因子可以直接调节脂肪细胞分化、代谢等生理过程。例如,研究证明PPARγ受体激活会引起前体脂肪细胞成脂分化从而导致肥胖;RXR激活可以促进脂肪分化和前脂肪细胞增殖^[8]^。一些具有内分泌干扰效应的化学物质往往可以通过调控这些关键内分泌核受体,从而表现出致肥胖效应,因此研究化合物与关键核受体的互作,探讨其对受体的结合、激活或拮抗效应,可以预测化合物的脂代谢干扰效应。

目前常用的研究化合物与核受体生物分子结合的研究方法包括分子对接分析、荧光偏振竞争结合实验等。分子对接通过计算模拟配体分子和受体大分子的相互作用,预测其亲和力和结合位点。荧光偏振竞争结合实验可以测定化合物与受体的结合力。荧光素酶报告基因实验常被用来研究化合物对受体大分子的结合转录激活效应,通过在表达该核受体的细胞模型中稳转/瞬转含有受体响应元件与荧光素酶报告基因的质粒,再利用荧光素酶检测系统,测定化合物对受体的激活或拮抗效应。利用分子互作研究模型,可以实现化合物对核受体作用的高通量筛选,从而初步预测环境致肥胖物质的脂代谢干扰潜力与分子作用靶点。

### 1.2 细胞成脂分化模型

离体细胞实验基于不同类型细胞构建离体分化实验模型,检测化合物对细胞中脂肪生成或代谢的影响。目前常用于脂代谢干扰效应的细胞模型比较多,其中小鼠3T3-L1细胞源自17~19天的小鼠胚胎,作为一种前体脂肪细胞,在化合物脂代谢研究中应用最为广泛^[8]^。其分化模型是采用包含胰岛素、地塞米松与3-异丁基-1-甲基黄嘌呤的诱导剂混合物进行诱导处理,细胞经过7~10天分化形成成熟的脂肪细胞^[10,11]^。此外,前体脂肪细胞模型还有3T3-F442A、NIH3T3-L1、小鼠骨髓来源基质前脂肪细胞系OP9、新生小鼠皮肤来源的WAT前脂肪细胞ST-13和永生化棕色前脂肪细胞UCP-1细胞系^[12]^。OP9细胞本身缺乏巨噬细胞集落刺激因子,在含有油酸与胰岛素的培养基中暴露72 h后,细胞内能够形成大量甘油三酯(TG)脂滴,且对PPARγ和RXR活性配体敏感,可用于快速评价化学物质对脂肪生成的影响^[13,14]^。考虑到使用鼠源模型开展的研究可能会因物种差异而难以用于人体健康效应评估,一些研究者也考虑采用人源前体脂肪细胞进行实验,如原代人前脂肪细胞、具有高脂肪分化能力的源于脂肪组织基质血管部分的Simpson-Golabi-Behmel综合征前脂肪细胞(SGBS)、来自吸脂手术后的原代皮下人类前脂肪细胞PCS-210-010和人脂肪肉瘤细胞系SW872等^[12]^。

近年来,源自脂肪组织的间充质干细胞(mesenchymal stem cells, MSCs),又名脂肪衍生干细胞(adipose-derived stem cells, ASCs)已被用作动物和人类前脂肪细胞模型的替代品。ASCs具有多能性,可以分化为脂肪细胞、肌细胞、骨细胞和软骨细胞。该模型的优点是可以评估化合物对脂肪细胞定向分化的影响及内在分子机制。该类型细胞模型主要有小鼠胚胎成纤维细胞C3H10T1/2、人脂肪干细胞hASCs以及鼠/人源的原代间充质干细胞^[12]^。由于体外单一类型细胞培养没有办法模拟人体内真实的环境,细胞间互作过程往往会被忽略。为了更好地模拟体内脂肪组织的微环境,以及致肥胖物质对不同器官影响的生理学过程,一些科学家还开发了3D细胞培养模型^[15]^,但这种实验存在成本高、实验操作复杂等问题。

离体细胞实验操作简单,经济有效,能够实现致肥胖物质的高通量筛选。然而,这类实验方法也存在一些缺陷,如不能完全模拟活体的系统生理学过程,无法探讨性别差异等因子的影响等^[10]^。

### 1.3 动物脂代谢实验模型

活体动物实验可以提供污染物致肥胖效应的更为系统可靠的毒理学数据,从而客观评估机体内分泌系统受到干扰、产生炎症、导致肥胖发生发展的全过程。啮齿动物小鼠是最常用的研究肥胖的动物模型,饲养方便、繁殖周期短,且其生物学和解剖学与人类具有一定相似性,研究结果对于人体健康效应评估具有重要参考意义。通过小鼠模型,可研究污染物对小鼠体重、血脂、脂肪组织累积、非酒精性脂肪肝(nonalcohol fatty liver disease, NAFLD)形成、代谢组等影响。研究常结合不同饮食条件,如高脂或高胆固醇(T-CHO)等,探讨致肥胖物质对实验动物脂代谢的影响,也可构建一些转基因肥胖小鼠模型如瘦素基因敲除小鼠(ob/ob小鼠)、瘦素受体基因敲除小鼠(db/db小鼠)等开展脂代谢研究。在脂代谢研究中,其他常见实验动物模型还包括大鼠、斑马鱼和非洲爪蟾等^[10]^。

虽然活体动物研究可以获得关于污染物脂代谢影响的重要毒理数据,但仍需要注意一些问题。例如,由于存在物种差异,基于动物实验获得的结果,如剂量反应、暴露窗口、慢性毒性影响等,不能完全推及人类。

## 2 双酚类化合物的脂代谢研究

BPA被广泛用于制造聚碳酸酯塑料和环氧树脂,作为抗氧化剂存在于各种塑料制品、饮料罐、食品包装材料等产品中,并经内层包装材料释放,污染由此保存的食品与饮料^[16]^。BPA可通过食物和饮用水,经消化道摄入机体,也可通过呼吸、垂直传播等途径暴露。根据2005年的一项研究显示,美国95%普通人群尿液样本中检出BPA^[17]^。2006年研究报道人体血清和母乳中BPA的暴露水平为0.3~5 ng/mL,约为1~20 nmol/L^[18]^。此外,人们在胎盘、脐带血、新生儿血液中也发现了BPA的存在^[19]^。考虑到BPA具有类雌激素效应,可对人体健康产生有害影响,目前许多BPA替代品已被开发并应用到工业生产中。常见的BPA替代品包括双酚B(BPB)、双酚F (BPF)、双酚S (BPS)、双酚E(BPE)、双酚AF(BPAF)等^[20]^。由于化学结构相似性,这些替代品同样可能具有潜在的毒性风险,表现出与BPA类似的毒性效应,如发育毒性、代谢干扰效应、免疫毒性等^[20,21]^。研究显示,BPA及其替代品可诱导细胞凋亡,且部分替代品的毒性效应大于BPA^[22]^。

### 2.1 脂肪生成和代谢

脂肪生成是多能干细胞或前体脂肪细胞定向分化形成成熟脂肪细胞的过程。致肥胖物质可以通过增加脂肪细胞的数量和大小来增加脂肪组织的累积,影响机体脂代谢。大量离体细胞实验表明BPA具有促成脂效应。低剂量BPA可促进3T3-L1前体脂肪细胞分化,诱导脂质积累,上调脂肪细胞分化标志物(*Pparγ*、*C/ebpα*和*Fabp4*)^[23,24]^。在原代大鼠脂肪细胞中也发现BPA的促脂质累积作用^[25]^。0.01~1 μmol/L BPA暴露诱导ASCs定向分化形成脂肪细胞,胞内TG含量增加,脂质生成相关基因(*Pparγ*、*C/ebpα*和*Lpl*)表达升高^[26,27]^。然而,10 μmol/L BPA可引起大鼠ASCs中DNA损伤、细胞凋亡、脂肪细胞分化降低,表现出细胞毒性效应^[26]^。关于BPA的促成脂效应也有不同的研究发现。例如,2018年De Filippis等^[28]^发现环境相关浓度BPA暴露主要引起胰岛素抵抗,减少蛋白激酶B(AKT)磷酸化,增加促炎水平,但对多种体外细胞系的脂肪生成并没有影响。此外,BPA的主要代谢物BPA葡萄糖醛酸(BPA-G)通常被认为没有生物活性,但10 μmol/L BPA-G可显著增加3T3-L1和人原代脂肪细胞的脂质积累,诱导脂肪形成分子标志物的表达^[29]^。BPA-G没有雌激素活性,但雌激素受体抑制剂氟维司群(ICI)可抑制BPA-G诱导的脂肪水平升高^[29]^。

离体实验证明,BPA类似物(BPB、BPE、BPF、BPS)也具有促成脂效应,是潜在的环境致肥胖物质^[23,24,30,31]^。研究显示,在3T3-L1成脂分化实验中,BPS与BPA在相同暴露浓度下,前者诱导的成脂分化基因(*Pparγ*、*C/ebpα*、*Perilipin*)表达水平更高,因此表现出更强的促成脂分化能力^[23,24]^。BPS诱导hASC以及来自女性供体的人原代皮下前体脂肪细胞分化,促进脂质积累和成脂分化分子标志物(*PPARγ*、*C/EBPα*、*LPL*、*FABP4*)转录表达的增加^[30]^,并且这种作用可能是通过直接激活核受体PPARγ介导的^[32]^。0.1 nmol/L低剂量BPAF显著增加了脂肪生成,而10 nmol/L BPAF则显著降低了脂肪生成^[33]^。四甲基双酚F(TMBPF)表现出抗脂肪形成的作用,0.01~0.1 μmol/L TMBPF暴露显著减少了30%~40%的脂质产生^[33]^。关于BPF,一些研究表明这种化合物低浓度暴露可以促进3T3-L1和hASCs细胞成脂分化^[31]^;然而Drobna等^[24]^研究发现,BPF暴露可降低脂肪分化晚期基因的表达,可能表现为脂质减少效应。还有研究显示,BPF暴露对3T3-L1脂肪细胞大小没有影响,但会降低瘦素、脂联素等脂肪因子的表达,干扰胰岛素信号通路^[34]^。化合物混合暴露在一定情况下可以更为客观反映真实环境暴露情况,有助于评价不同致肥胖物质之间的相互作用及由此导致的脂代谢干扰作用。例如研究BPA及其替代物对hASCs的混合暴露发现,细胞成脂分化过程与脂质含量同样呈现出暴露剂量依赖性促进与增加,且雌激素受体抑制剂可以显著抑制该效应,提示雌激素受体介导的信号通路参与了化合物促成脂效应^[35]^。

上述化合物的离体研究发现同样也有活体实验证据支持。例如,20~500 μg/L BPA暴露可引起成年雄性斑马鱼食欲亢进和肥胖,其中大麻素受体1型(CB1)激活发挥了重要作用^[36]^。围产期接触BPS(100 ng/(g(bw)·d))可干扰雄性小鼠后代脂肪和葡萄糖代谢,显著增加仔鼠体重、肝脏和附睾白色脂肪组织重量、血清丙氨酸氨基转移酶(ALT)活性、肝脏TG和总胆固醇(TC)含量^[37]^,并且BPS可以引起小鼠多代肥胖效应^[38]^。基于动物实验可以很好地探讨化合物致肥胖效应的性别差异。研究显示,妊娠期BPA、BPS暴露会增强女性而非男性前脂肪细胞的分化能力,说明这些化合物的致肥胖效应具有性别差异性^[39]^。

除了毒理学实验外,流行病学资料也显示BPA暴露与肥胖风险有关。一项基于1093名参与者的前瞻性队列研究发现,血清BPA水平与TC、低密度脂蛋白(LDL-C)、非高密度脂蛋白水平呈正相关^[40]^。BPA每增加1 ng/mL,肥胖风险就会增加11%(比值比(OR): 1.11; 95%置信区间(95% CI): 1.10~1.13)^[41]^。这些研究表明BPA暴露与人群肥胖发生风险呈正相关。

### 2.2 肝组织脂代谢

肝脏参与维持机体能量代谢,也是激素合成和外源化合物代谢的重要场所。机体脂代谢紊乱,可以造成NAFLD的形成与进展。一些EDCs一方面可在肝脏中富集代谢,另一方面也可能会对肝细胞的脂代谢产生干扰,从而引起肝脏脂肪变性与肝损伤。研究显示,低浓度BPA暴露会改变肝脏中的脂代谢,影响脂质相关转录因子表达、炎症和线粒体功能障碍,并促进肝脏中脂质积累^[42,43]^。BPA对肝脏脂质的影响可能与Kupffer细胞向促炎M1表型极化有关^[44]^。研究还发现,BPA暴露可以增加HUH-7细胞内活性氧(ROS)水平,并通过上调游离脂肪酸摄取转运蛋白CD36来促进脂肪酸摄取^[45]^。此外,在高脂肪/高胆固醇/高胆汁酸饮食(HFCCD)条件下,BPA (50 mg/kg)暴露8周的C57BL/6小鼠出现脂肪性肝炎样表型,表现为肝纤维化指标*α*-平滑肌肌动蛋白(*α*-SMA,)、凋亡指标剪切型半胱天冬酶3(cleaved caspase 3)、氧化应激指标8-羟基脱氧鸟苷(8-OHdG)升高^[45]^。BPA长期暴露(0.5 μg/(kg·d), 10个月)能够显著促进雄性小鼠肝脏TG和TC积累,这与参与脂代谢的基因甲基化改变有关^[46]^。不同剂量(50 μg/L、5000 μg/L)的BPA暴露4周后可以干扰鸡的脂代谢并引起肝脏炎症与铁死亡,具体表现为BPA显著增加TG、TC和LDL-C的含量,降低高密度脂蛋白胆固醇(HDL-C)水平,改变参与脂肪酸*β*-氧化(*Ampkα*、*Cpt*-*1*和*Pparα*)、合成(*Acc*、*Fas*、*Scd-1*和*Srebp*-*1*)和吸收(*Lpl*和*Cd36*)的基因水平^[47]^。BPA暴露还可以改变炎性因子表达,引起小鼠肝脏中白细胞介素-1β(IL-1β)、白细胞介素-18(IL-18)、肿瘤坏死因子-α(TNF-α)水平明显升高,铁含量提高,脂质过氧化相关基因(*Lpcat3*、*Acsl4*和*Alox15*)表达增加,抗氧化系统相关基因(*Gpx4*、*Slc7a11*和*Slc3a2*)表达降低,表现为铁死亡^[47]^。G蛋白偶联雌激素受体(GPER)参与了BPA引起的肝毒性效应^[47]^。此外,孕期作为敏感窗口期,极易受到外源污染物暴露的干扰。研究发现产前BPA暴露能够诱导子代小鼠脂肪肝的发生^[48⇓⇓⇓-52]^。

基于水生生物的研究同样显示BPA具有致肥胖作用。BPA暴露可引起成年雄性稀有米诺鱼肝脏脂质沉积显著增加,TG水平升高1.84~22.87倍,但伴随着甘油二酯水平的降低, TG合成相关基因表达上调,其降解相关基因表达下调。BPA暴露可干扰TG转运相关基因的表达^[53]^。BPA急性或慢性暴露均可诱导斑马鱼肝脏脂质堆积与脂肪变性,这与脂肪合成、摄取增加以及脂质分解抑制有关^[54]^。环境相关浓度BPA暴露120天后,雌鱼肝脏中脂质转运和合成增加,高浓度BPA处理后斑马鱼的整体代谢水平提高,且BPA对雄鱼的脂质沉积表现出剂量依赖性,脂代谢相关基因表达受到影响^[55]^。

研究表明,BPA替代物也可引起肝脂代谢紊乱。例如,BPF和BPAF暴露会降低HFD小鼠肝脏中甘油酯类化合物和TC水平^[56]^。斑马鱼从胚胎期开始暴露于含BPF、BPAF(0.5 μg/L)的水溶液中,60天后,实验鱼出现肝脏脂肪变性和胰岛素抵抗^[57]^。BPS暴露120天可诱导雄性斑马鱼肝脏TG和TC水平升高,血浆天冬氨酸转氨酶(AST)和ALT活性升高,内质网应激引起的未折叠蛋白反应可能是BPS调控脂代谢产生毒性效应的作用机制^[58]^。这些研究表明,BPA替代物并不安全,其大量生产应用可能会引入新的环境健康风险。表1总结了BPA及其类似物在脂代谢效应方面的相关研究。

**表1:** 双酚A及其类似物的脂代谢干扰效应研究

Chemical	Test tissue	Model	Effects	Mechanisms
BPA	adipocytes	3T3-L1	↑ lipid storage^[23,24,59,60]^	lipogenesis^[23,24]^; PPARγ signal^[59]^; inhibiting autophagosome-lysosome fusion^[60]^
			insulin resistant^[28,61,62]^	inflammation^[28,62]^; JNK signal^[62]^
		C3H10T1/2	— adipogenesis^[28]^	
		rASCs	↑ adipose differentiation^[26]^	
		hASCs	↑ lipid storage^[27]^	lipogenesis^[27]^
		zebrafish	↑ lipid storage, ↑appetite^[36]^	CB1 signal^[36]^
	hepatic tissue	HepG2	↑ lipid storage^[44]^	inflammation, ER signal^[44]^
		HUH-7	↑ lipid storage^[45]^	oxidation^[45]^
		mouse	↑ lipid storage^[44⇓-46]^	inflammation^[44]^; oxidation^[45]^; DNA methylation patterns^[46]^
		zebrafish	↑ lipid storage^[54,55]^	lipogenesis, ↓TG degradation^[54]^
		male Gobiocypris rarus	↑ lipid storage^[53]^	lipogenesis, ↓TG degradation^[53]^
		chicken	↑ lipid storage, ferroptosis^[47]^	fatty acid synthesis/uptake, inflammation, GPER signal^[47]^
BPA-G	adipocytes	3T3-L1 and primary human preadipocytes	↑ lipid storage, ↑ adipose differentiation^[29]^	non-classical ER transcriptional activation^[29]^
BPAF	adipocytes	hASCs	↑ adipogenesis at a low dose^[33]^	
	hepatic tissue	mouse	↓ lipid storage^[56]^	PPAR signal^[56]^
BPB	adipocytes	3T3-L1	↑ lipid storage^[31]^	
BPE	adipocytes	3T3-L1	↑ lipid storage^[31]^	
BPF	adipocytes	3T3-L1	↑ lipid storage^[23,31]^	lipogenesis^[23]^
			— adipogenesis, ↓ insulin-stimulated glucose metabolism^[34]^	inhibiting IRS-1/PI3K/AKT pathway^[34]^
			↓ differentiation genes expression^[24]^	
			↓ expressions of leptin, adiponectin and apelin^[34]^	
		hASCs	↑ lipid storage, ↓ adipogenic markers^[30]^	ER signal^[30]^
	hepatic tissue	mouse	↓ lipid storage^[56]^	PPAR signal^[56]^
		zebrafish	↑ lipid storage^[57]^	intestinal cell heterogeneous response^[57]^
BPS	adipocytes	3T3-L1	↑ lipid storage^[31]^	
		primary human preadipocytes	↑ lipid storage, ↑ adipose differentiation^[32]^	PPARγ, GR signal^[32]^
		hASCs	↑ lipid storage^[30]^	
		perinatal exposure in mouse	↑ weight, ↑ TG and T-cholesterol accumulation^[37]^	inflammation^[37]^
			multigenerational obesogenic effect^[38]^	
		zebrafish	↑ lipid storage^[63]^	De novo synthesis^[63]^
	hepatic tissue	zebrafish	↑ TG, T-cholesterol accumulation, ↑ ALT, AST^[58]^	endoplasmic reticulum stress response^[58]^
TMBPF	adipocytes	hASCs	↓ adipogenesis, cytotoxicity^[33]^	

↑: increase; ↓: decrease; —: no effect; BPA: bisphenol A; BPA-G: bisphenol A *β*-D-glucuronide; BPAF: bisphenol AF; BPB: bisphenol B; BPE: bisphenol E; BPF: bisphenol F; BPS: bisphenol S; TMBPF: tetramethyl bisphenol F; rASCs: rat adipose-derived stem cells; hASCs: human adipose-derived stem cells; TG: triglyceride; ALT: alanine aminotransferase; AST: aspartate aminotransferase; PPAR: peroxisome proliferator-activated receptor; JNK: Jun N-terminal kinase signaling pathway; CB1: cannabinoid receptor type 1; ER: estrogen receptor; GPER: G protein-coupled estrogen receptor; IRS: insulin receptor substrate; PI3K: phosphoinositol 3-kinase; AKT: protein kinase B; GR: glucocorticoid receptor.

## 3 烷基酚类化合物的脂代谢研究

烷基酚类化合物(alkylphenol compounds, APs)由一个苯酚环和一个烷基取代基组成,是原油中的一类重要成分,常用于制备酚醛树脂、聚合物、热稳定剂、抗氧化剂和固化剂^[64]^。APs可与环氧乙烷结合生成烷基酚聚氧基酸酯(APEs),这是一类非离子表面活性剂,广泛用于洗涤剂、润湿剂、乳化剂、增溶剂和发泡剂以及油漆、农药和除草剂中^[65]^。APEs在降解过程中会产生更具有持久性和亲脂性的APs,如壬基酚(NP)和辛基酚(OP)^[66]^。APs通过工业生产的各环节或生活垃圾排放到环境中,主要污染水环境,特别是海洋环境。海水和海产品中常检测到APs,其中4-NP检出率高、分布广泛,平均含量为0.02~2.76 μg/L^[67]^。此外,在空气、土壤、沉积物和生物样本中也有NP和OP检出^[66]^。毒理学研究显示,APs具有类雌激素效应,且其雌激素活性与化学结构高度相关,是潜在的内分泌干扰物与环境致肥胖物质^[64,65]^。

### 3.1 脂肪生成和代谢

4-NP是一种具有类雌激素效应的内分泌干扰物,研究显示它同样具有促脂肪生成作用,被鉴定为致肥胖物质^[68⇓-70]^。基于C3H10T1/2细胞分化模型的实验表明,4-NP可显著上调脂肪生成标志物PPARγ和FASN mRNA/蛋白的表达,增加脂肪生成,促进细胞成脂分化^[68,69]^。4-NP暴露大鼠体重增加显著,血清TG、TC和LDL-C含量高于对照组^[70]^。小鼠围产期暴露于4-NP会影响后代脂肪形成,增加后代体重、血清TC和葡萄糖水平^[71]^,并且这种效应可以遗传到F2代^[72]^。

由于APs类化合物的化学结构具有高度相似性,除4-NP外,其他APs化合物也可能同样具有促成脂作用,但目前相关研究证据并不多。有限的文献数据显示,4-叔辛基苯酚(4-t-OP)可抑制小鼠胚胎结缔组织细胞系C3H10T1/2细胞的成骨分化,促进细胞系向脂肪细胞分化,增加PPARγ蛋白表达、TG和脂联素含量^[73]^。4-己基苯酚(4-HP)可激活脂肪生成相关基因的表达,促进前脂肪细胞3T3-L1的成脂分化^[74]^。2,4-二叔丁基酚(2,4-DTBP)暴露可引起hMSCs脂质积累和脂肪生成标记基因表达增加,该过程是通过激活RXRα信号通路引起PPARγ/RXRα异源二聚体的增加^[75]^。2,4,6-三叔丁基酚被发现能够显著诱导人胎盘绒毛膜癌细胞系JEG-3细胞内TG积累。此外,HP可显著诱导JEG-3细胞ROS产生,抑制胎盘芳香酶活性,并以暴露剂量依赖性方式诱导多不饱和脂质增加,而4-十二烷基酚则可以增加细胞内磷脂酰胆碱(PCs)和TG的合成^[76]^。

### 3.2 肝组织脂代谢

肝脏胰岛素信号通路可受到APs化合物暴露的影响。大鼠经口暴露于NP(15、150和1500 mg/kg)45天后,肝脏中胰岛素信号分子胰岛素受体(IR)、IR底物(IRS-1、IRS-2)和磷脂酰肌醇-3激酶的蛋白质水平下降,H_2_O_2_水平增加,抗氧化酶的活性降低,表明NP可诱导ROS产生和氧化损伤,下调肝脏中的胰岛素信号传导^[77]^。高蔗糖高脂肪饮食的小鼠受到NP暴露后会出现肝脏脂肪变性,炎症细胞浸润以及脂肪生成基因上调,最终导致非酒精性脂肪肝形成^[78]^。基于离体实验的研究数据表明,4-HP可以通过暴露剂量依赖的方式促进肝脂形成,这与肝细胞对外源性脂质油酸(OA)的摄取增加有关,雌激素受体拮抗剂ICI可有效阻断4-HP引起的肝脂累积效应,表明ER信号通路可能在该过程中发挥了重要作用。

## 4 合成酚类抗氧化剂的脂代谢干扰研究

合成酚类抗氧化剂(SPAs)是一类人工合成的化学品,由于其生产成本低、抗氧化性能好,被广泛添加在食品、化妆品、塑料等各种工业产品中。目前在食品中允许使用的SPAs包括丁基化羟基苯甲醚(BHA)、丁基羟基甲苯(BHT)、没食子酸丙酯(PG)和叔丁基对苯二酚(TBHQ)^[79,80]^。BHA是两种同分异构体3-BHA与2-BHA的混合物。另外,没食子酸辛酯(OG)和没食子酸十二酯(DG)也常被用于合成抗氧化剂。联合国粮农组织/世卫组织食品法典委员会的联合报告规定,单独或与其他合成抗氧化剂组合的食品中SPAs的最大允许含量为200 mg/kg^[81]^。在一些干谷物、熟食(经煮沸或油炸的食品、甜品等)以及饮料中常可检测到BHA的存在。另外,BHA与其他抗氧化剂具有协同作用,所以经常会混合添加于食品中,以期获得更好的抗氧化性能^[79]^。然而,毒理学资料显示,SPAs具有潜在的肝毒性、内分泌干扰作用甚至致癌性^[79,80]^,因此可能存在健康风险。

### 4.1 脂肪生成和代谢

研究显示,BHA中的3-BHA可以通过调节脂肪生成生物标志物的转录和蛋白表达,从而促进前体脂肪细胞3T3-L1成脂分化与胞内脂质累积,且化合物暴露的有效窗口期为细胞分化前4天^[81]^。与3-BHA不同,同分异构体2-BHA并不表现出促成脂效应。3-BHA暴露可显著降低分化早期(G0/G1期)细胞群,增加S期细胞群,促进细胞增殖,干扰PPARγ信号通路上游事件,诱导细胞分化并促进脂质合成^[81]^。活体动物实验也表明3-BHA对机体脂肪组织累积具有显著影响。3-BHA长期暴露,可调控脂肪生成、脂代谢和炎症功能相关基因转录水平,引起小鼠体重、血脂增加和白色脂肪组织积累,但对糖代谢和胰岛素敏感性没有产生影响。与正常饮食小鼠相比,高脂饮食小鼠更容易受到3-BHA的暴露影响,出现的脂质累积效应更加显著^[82]^。基于C3H10T1/2细胞定向分化模型的研究发现,在BMP7处理条件下细胞可以向棕色细胞分化,产热相关基因被显著诱导。然而,3-BHA暴露后可以有效促进C3H10T1/2细胞分化,导致胞内脂质积累,脂肪生成标志物(*Pparγ*、*Adiponectin*、*Fabp4*等)表达升高。然而,与对照组相比,3-BHA处理后并没有诱导线粒体、产热相关基因的表达增加,表明3-BHA引起C3H10T1/2细胞棕脂分化表型向白色脂肪转变,其分子调控机制为smad1/5/8磷酸化^[83]^。

### 4.2 肝组织脂代谢

研究显示,BHA可以影响大鼠肝脏能量代谢,降低肝脏糖异生,增加糖原分解、糖酵解,降低ATP水平,促进ROS生成^[84]^。高脂饮食条件下小鼠经口服暴露于10 mg/kg 3-BHA 18周后,肝脏TG浓度显著高于对照组,脂肪变性严重,脂质组学分析表明,在HFD条件下3-BHA处理引起鞘脂、甘油磷脂和甘油酯等30种脂质水平发生显著变化^[85]^,说明3-BHA长期暴露可改变小鼠肝脏脂代谢稳态,并加剧高脂饮食诱导的NAFLD的形成。离体细胞实验也显示50 μmol/L 3-BHA暴露条件下,HepG2细胞对OA的摄取增加,导致胞内TG累积^[85]^,这一结果很好地验证了活体动物实验的发现。

### 4.3 肾组织脂代谢

肾脏是一个代谢高度活跃的器官,脂代谢障碍会诱发肾脏疾病的发生和进展,如透明细胞肾癌。关于3-BHA对肾脏脂质稳态的影响仍不确定。基于人肾HK-2细胞的实验发现,3-BHA暴露可显著降低HK-2细胞内的脂质积累,并且呈现暴露浓度与暴露时间依赖方式。这种化合物主要通过抑制细胞对葡萄糖的吸收,加速糖酵解过程,从而导致胞内脂质含量降低,该结果也得到代谢组学数据的支持。分子机制研究显示,3-BHA对AR具有拮抗作用,从而可降低细胞内脂肪从头生成,引起胞内脂质减少^[86]^。3-BHA降低肾细胞脂质累积的效应,与其促脂肪生成及肝脂累积效应相反^[81⇓-83,85]^,提示机体不同组织器官对环境污染物暴露可产生显著不同的响应,值得未来研究进一步探索。

## 5 酚类化合物干扰脂代谢的毒理机制

环境致肥胖物质调控脂代谢的毒理机制非常复杂,其中,关于关键内分泌核受体(nuclear receptors, NRs)的转录激活调控、表观遗传改变等的研究^[87]^相对较多。

### 5.1 核受体

在脂肪细胞生成及脂代谢过程中,一些核受体起着重要的调控作用。双酚类化合物被报道可与许多NRs,如PPARγ、ER、GR等,结合并激活响应的信号通路,这与该类化合物对3T3-L1细胞、前脂肪细胞以及活体小鼠的促脂肪生成作用有关^[30,32]^。APs作为一类典型类雌激素化合物,可结合并激活雌激素受体,这在APs诱导的脂质积累过程中也发挥着重要的作用^[74]^。3-BHA干扰肾脂代谢过程则与化合物具有AR拮抗活性有关^[86]^。

### 5.2 炎症和氧化应激

在脂肪组织中,致肥胖物质可以促进炎症状态并增加氧化应激。致肥胖物质可以激活促炎途径,上调细胞因子如白细胞介素-6(IL-6)、IL-1β、TNF-α的表达和释放^[37,57,87]^。BPA及其替代品可诱导巨噬细胞的M1极化,从而维持白色脂肪细胞群^[88]^。BPA已被证明可以促进成熟脂肪细胞的氧化应激并增加ROS水平,从而导致不同脊椎动物的脂质氧化增强。用活性氧清除剂*N*-乙酰半胱氨酸(NAC)治疗可改善BPA诱导的肝脏脂质积累和脂肪性肝炎^[45]^。此外,烷基酚可显著诱导ROS的产生并抑制胎盘芳香化酶活性,从而导致脂质积累增加^[76]^。

### 5.3 肠道微环境

机体肠道微环境与肥胖发生密切相关。研究发现,在无菌小鼠体内移植肥胖个体的肠道微生物群落,可以诱导受体小鼠肥胖^[89]^,说明肠道微生物在机体脂代谢与肥胖形成过程具有重要作用。一些环境致肥胖物质可以干扰肠源性因子,从而影响机体脂代谢。例如,双酚A及其类似物可促进肠道嗜铬细胞产生5-羟色胺,干扰机体代谢^[90]^。这类化合物还可以诱导肠道细胞异质变化,激活肠细胞对脂质的摄取和吸收^[57]^。

### 5.4 表观遗传

近年来表观遗传修饰在调节脂肪组织基因表达的作用方面引起了人们的高度关注。一些体外实验研究显示,BPA、BPS和BPF暴露可引起*microRNA-26a* (*miR-26a*)基因下调^[91]^,而*miR-26a*和*miR-26b*对脂肪生成至关重要,被证明是脂肪细胞褐变过程的关键调节分子^[92]^。BPA诱导肝细胞脂质累积,也与参与脂代谢基因的表观遗传重编程有关,如DNA甲基化水平发生改变。暴露于BPA后的小鼠肝脏中DNA甲基化转移酶表达水平降低,导致脂质合成相关基因*Srebf1*和*Srebf2*的DNA甲基化水平降低,其转录表达水平增加^[46]^。

### 5.5 其他信号通路

环境致肥胖物质还能够通过其他非激素信号通路调控脂代谢,如干扰生长因子下游的受体激酶通路、神经递质通路、发育信号通路等^[8]^,从而改变食欲、饮食偏好,影响机体对脂质的摄取^[93]^。研究发现,大鼠生命早期接触BPA会改变突触前后信号通路、促进成瘾和强迫行为、增加饮食摄入,由此引起肥胖发生^[94]^。3T3-L1细胞暴露于BPA会干扰脂肪因子的分泌、增加瘦素水平,提示这种化合物可能对食欲具有调控作用^[95]^。

## 6 研究展望

在过往近20年的研究工作基础上,人们虽然已经识别并鉴定了一些致肥胖物质及其毒理效应,如多种SPCs等,但客观而言,我们对环境致肥胖物质的认识仍是冰山一角,需要更多研究来发现环境中新的致肥胖物质,并解析它们的毒性作用方式。此外,基于单一的筛选实验或分析方法,不能全面有效地揭示致肥胖物质的致毒效应,亟需基于计算建模、多靶点离体分析技术与活体动物实验构建污染物致肥胖效应的综合测试体系,有望为寻找并发现环境中新的致肥胖物质提供可靠的技术支持。在此基础上,结合流行病学研究可以客观评价可疑污染物的健康危害。还有,除了遗传因素外,肥胖易感性还可受到环境因素重编程,因此需要综合与营养、活动、压力、感染、微生物等一起开展研究,以准确评估环境对肥胖的影响。最后,需要考虑的是,环境化学物质的毒理效应受暴露时间、暴露敏感窗口期、暴露模式等影响,关于致肥胖物质的关键分子靶标,性别二态性,表观遗传机制和多代、跨代效应,我们还知之甚少。未来研究尚需针对以上这些方面开展更多工作,以期获得对环境致肥胖物质更为客观全面的认识,从而服务于健康风险的正确评价与健康危害的预防。
